# Fast-feedback for unattended data collection at Diamond Light Source

**DOI:** 10.1063/4.0000946

**Published:** 2025-10-27

**Authors:** Nicholas Devenish, James Beilsten-Edmands, Dimitrios Vlachos

**Affiliations:** Diamond Light Source, Didcot, Oxfordshire, United Kingdom

## Abstract

Diamond Light Source's I03 beamline now spends most of its time in "Unattended Data Collection" mode. To maximise throughput, it is critical that as little time is spent waiting for x-ray centering as possible. In this talk, I will describe the GPU-based fast-feedback service that we have built, how we integrated it into our wider acquisition and analysis systems, and how we plan to evolve it for future needs.

